# Fast probabilistic file fingerprinting for big data

**DOI:** 10.1186/1471-2164-14-S2-S8

**Published:** 2013-02-15

**Authors:** Konstantin Tretyakov, Sven Laur, Geert Smant, Jaak Vilo, Pjotr Prins

**Affiliations:** 1 Institute of Computer Science, University of Tartu, J. Liivi 2, 50409 Tartu, Estonia; 2 Laboratory of Nematology, Wageningen Agricultural University, Droevendaalsesteeg 1, 6708 PB Wageningen, The Netherlands; 3 Groningen Bioinformatics Center, University of Groningen, P.O. Box 14, 9750 AA Haren, The Netherlands

## Abstract

**Background:**

Biological data acquisition is raising new challenges, both in data analysis and handling. Not only is it proving hard to analyze the data at the rate it is generated today, but simply reading and transferring data files can be prohibitively slow due to their size. This primarily concerns logistics within and between data centers, but is also important for workstation users in the analysis phase. Common usage patterns, such as comparing and transferring files, are proving computationally expensive and are tying down shared resources.

**Results:**

We present an efficient method for calculating file uniqueness for large scientific data files, that takes less computational effort than existing techniques. This method, called *Probabilistic Fast File Fingerprinting (PFFF)*, exploits the variation present in biological data and computes file fingerprints by sampling randomly from the file instead of reading it in full. Consequently, it has a flat performance characteristic, correlated with data variation rather than file size. We demonstrate that probabilistic fingerprinting can be as reliable as existing hashing techniques, with provably negligible risk of collisions. We measure the performance of the algorithm on a number of data storage and access technologies, identifying its strengths as well as limitations.

**Conclusions:**

Probabilistic fingerprinting may significantly reduce the use of computational resources when comparing very large files. Utilisation of probabilistic fingerprinting techniques can increase the speed of common file-related workflows, both in the data center and for workbench analysis. The implementation of the algorithm is available as an open-source tool named pfff, as a command-line tool as well as a C library. The tool can be downloaded from http://biit.cs.ut.ee/pfff.

## Background

A rapid increase in data generation by recent high-throughput acquisition technologies for genomics, transcriptomics and metabolomics raises new challenges in data handling and analysis. Data warehouses containing petabytes-worth of biological data are increasingly common [1]. At this scale, routine tasks of data management such as storage, backup, transfer and synchronization of files become increasingly problematic.

For example, a typical next-generation sequencer, such as the Illumina Genome Analyzer II system, produces approximately 1 terabyte of data per single ten-day-long run, which must be moved from the capture workstation to the analysis resource [2]. Other systems have similar data yields. The transfer of one terabyte over a Gigabit Ethernet network connection takes more than two hours. A more typical connection of 10-100 Mbit, like ones between geographically distant universities, requires between one and ten *days *to complete the transfer, assuming there is no other use of the network.

A common usage pattern is to replicate file collections at multiple locations for mirroring and backups, therefore files are moved around to keep copies synchronised. A number of techniques exist for performing file synchronization in a smart manner, avoiding transfers whenever the files are proven to be identical on both ends [3-6].

With these methods, file similarity is tested by calculating a *hash value *over the full file using a particular algorithm. This hash value is a number, typically sized between 64 and 2048 bits, such that the chances of obtaining the same hash value for two distinct files - the situation referred to as a *collision *- are negligibly small, on the order of 2^-64 ^to 2^-2048^. This way, hash values act like a fingerprinting technique, which allows to compare files at two sites without actually transferring them.

However, with data volumes exceeding terabytes, even scanning the files to compute conventional hashes or fingerprints is already expensive. Indeed, the time required to scan a terabyte of data is about 25 minutes even for a modern high-speed 6Gbps drive. The situation is made worse by the fact that in many data centers disks are a shared resource. Consequently, a file synchronization method that is based on full file access can be intolerably slow for this setting.

There are ways of alleviating the problem by storing the precomputed hashes together with data files or tracking file changes on a system level. However, as of today, not many public data warehouses do it consistently. Moreover, biological data sets are often gathered by independent authorities, such as Array Express [7] and the NCBI Gene Expression Omnibus (GEO) [8]. As a result, these collections may contain a number of files with equal content that bear different filenames, or, contrarily, have equal filenames, but differ in content. If file fingerprinting is not supported by download servers, then any third party willing to download a consistent union of all the files has no other choice but to download all the files every time, even if many of them later turn out to be duplicates.

As a solution, we offer a new hashing algorithm, *Probabilistic Fast File Fingerprinting (PFFF)*, that computes file fingerprints by sampling only a few bytes from the file in a pseudorandom fashion. This makes on-demand hashing tractable. Most importantly, it can be applied over the network to quickly obtain hashes of files stored in remote third-party warehouses.

Although our approach might seem unorthodox, we demonstrate in the following sections that due to inherent variability in large-scale biological data, the risk of false positives due to sampling is negligible. In addition, we measure the performance of our approach on several storage and data access technologies. We discover that the performance gains due to sampling can vary considerably depending on the underlying technology. While PFFF outperforms conventional hashing for most file sizes, when used via web and on Flash storage, for hashing over the NFS network protocol, the benefits surface only with strict variability thresholds or at very large file sizes.

## Methods

### Standard file fingerprinting algorithms

Hashing is a common method to speed up file comparison. A hash function compresses files into short digests *(fingerprints) *that are much easier to compare. If two fingerprints are different, the corresponding files are different, and whenever the fingerprints are the same, the files are the same, except for a negligibly small probability of a *collision*, i.e., two different files having the same fingerprint.

Hash functions can be divided into two classes: deterministic and probabilistic. A *deterministic *hash function is simply a fixed algorithm *f *that maps a file to its fingerprint:

$$f:M\to {\left\{0,1\right\}}^{\ell },$$

where M  is the space of all possible files of interest and ℓ is the length of the hash. Examples are the well-known MD5 [9] or SHA-256 [10] algorithms. As the number of possible fingerprints is finite and the number of possible files is, in principle, infinite, there may exist infinitely many files with colliding fingerprints. Thus there is no formal guarantee that for *any *given pair of files deterministic hashes would only collide with a negligible probability. Still, finding collisions for cryptographic hash functions such as SHA-256 is believed to be extremely difficult, and hence the probability of accidental collisions is negligible for all practical purposes [11].

A *probabilistic *hashing scheme is not a single hash function but rather a *family *of functions

$$F=\left\{f_{k}:M\to {\left\{0,1\right\}}^{\ell }|k\in K\right\},$$

where K  is the *key space*. Such a function family is useful in file fingerprinting if for any two fixed files $x_{1},x_{2}\in M$, and a *randomly selected key k*, the probability of a collision is bounded by a small number *ε_c_*:

$$\text{Pr}\;\left[k\leftarrow K:f_{k}\left(x_{1}\right)=f_{k}\left(x_{2}\right)\right]\leq \varepsilon_{c}.$$

Function families that satisfy this condition are usually referred to as *ε*_c_-*almost universal*. Efficient algorithms for such *universal hash functions *have been known for decades. See [11,12] for further details.

### Inherent variability of biological data

Due to long research history, standard file fingerprinting methods have reached perfection and it is extremely difficult to outperform them if no additional assumptions can be made about the data. However, biological data collections are quite specific. In particular, the situation when two large data files meaningfully differ in only a few bits is very unlikely in practice. Empirical examination of large repositories of biological data confirms that whenever two differently named files coincide in more than 20% of bytes, they are either misformattings of the same data, or may be treated as equivalent for the purposes of most large-scale analyses. For compressed data files the similarity bound is as large as 90%. See Results for further details.

This observation can be postulated as the *δ-variability assumption*. Assume that each file is represented as a sequence of blocks, e.g. bytes. Then a data collection satisfies *δ*-variability assumption if any two distinct files of the same length *x *= (*x*_1_, . . . , *x_m_*) and *y *= (*y*_1_, . . . , *y_m_*) differ at least in *δ*-fraction of the blocks:

$$|\left\{i:x_{i}\neq y_{i}\right\}|\;\geq \;\delta m.$$

### New file fingerprinting method

Explicit use of data variability is the key to more efficient hashing. Let *x *and *y *be two files of length *m *satisfying the *δ*-variability assumption. Now, if we sample uniformly with replacement ℓ indices *i*_1_, . . . , i_ℓ _∈ {1 , . . . , *m*}, then the probability that all the corresponding blocks in two files coincide (a *sampling collision*) is bounded from above:

$$\varepsilon_{\text{s}}=\text{Pr}\;\left[x_{i_{1}}=y_{i_{1}},\;.\;.\;.\;,x_{i_{\ell }}=y_{i_{\ell }}\right]\leq {\left(1-\delta \right)}^{\ell }.$$

More generally, for any collection of *n *distinct files that satisfies the *δ*-variability assumption, the probability that a sample of ℓ blocks coincides for at least one pair of files is bounded by:

$$\varepsilon_{\text{fail}}=\text{Pr}\;\left[\text{Some}-\text{collision}\right]\leq 0.5n\left(n-1\right)\varepsilon_{\text{s}}.$$

For instance, if the variability threshold is *δ *= 0.5 and we sample 103 random blocks, a sampling collision for a single pair of files may emerge with probability *ε*_s _≤ 2^-103^. For a set of 10^6 ^files, some collision occurs with probability *ε*_fail _≤ 5 · 10^-20^, which is comparable to the probability of a hardware failure.

In general, any desired upper bound for collision probability *ε*_fail _can be achieved with

(1) $$\ell \geq \frac{\text{log}\left(1/\varepsilon_{\text{fail}}\right)+2\text{log}\;n}{-\text{log}\left(1-\delta \right)}$$

samples. Observe that ℓ does not depend on the file size. Also note that ℓ scales logarithmically with 1/*ε*_fail _and *n*, which means that improvements to the desired failure probability *ε*_fail _and the number of files *n *only moderately increase the required sample size.

Samples $\hat{x}=x_{i_{1}}...x_{i_{\ell }}$ and $ŷ=y_{i_{1}}...y_{i_{\ell }}$ do not have to be compared directly. Instead, we can further compress them by some universal hashing scheme $F=\left\{f_{k}\right\}$ and compare $f_{k}\left(\hat{x}\right)$ and $f_{k}\left(ŷ\right)$. As a result, the probability of collisions increases, because with some probability $f_{k}\left(\hat{x}\right)=f_{k}\left(ŷ\right)$ even if $\hat{x}\neq ŷ$. However, if F  is *ε*_c_-almost universal, this probability is bounded by *ε*_c _and consequently the overall probability of a single collision is only marginally increased:

$$\text{Pr}\;\left[f_{k}\left(\hat{x}\right)=f_{k}\left(ŷ\right)\right]\leq \varepsilon_{s}+\varepsilon_{c}.$$

Finally, note that all random bits needed to generate indices *i*_1_, . . . , *i*_ℓ _and the key *k *can be replaced with the output of a pseudorandom number generator. In practice, any sufficiently complex pseudorandom generator will be adequate. We choose to use the fast and currently widely popular Mersenne twister algorithm [13] in our implementation.

## Results

### Variability in biological data

The cornerstone of our new hashing algorithm is the assumption that any two files in a biological data collection can either be treated as identical or necessarily differ in at least a *δ*-fraction of places. To study to what extent this variability assumption holds for biological data, we tested several kinds of common biological datasets, including both DNA sequence and tabular numeric data, both in compressed and uncompressed forms (Table 1).

**Table 1:** Variability in biological data

Dataset	Description	File type	Number of files	Total size (in GB)	File size (in MB)		*δ*
						
					Min	Max	
E61/dat	Ensembl v61 genome annotation (DAT) and DNA sequence (FASTA) files in both compressed (gzip) and uncompressed forms.	dat	5544	169.57	5.04	1385.14	0.782
		
E61/dat.gz		dat.gz	5544	42.92	1.02	400.21	0.996
		
E61/fa		fa	1484	498.51	3.47	13306.96	0.015
		
E61/fa.gz		fa.gz	1484	95.25	1.0	973.15	0.594

GPL570/cel	Microarray files for the HG U133 Plus chip from GEO (all files of GPL570 platform as of 03.2011). Affymetrix CEL and CHP format files, in compressed (gzip) and uncompressed form.	cel	59892	1022.29	1.92	173.27	0.000
		
GPL570/cel.gz		cel.gz	59892	330.09	1.13	48.84	0.000
		
GPL570/chp		chp	2535	63.30	1.67	36.50	0.209
		
GPL570/ch.gz		chp.gz	2535	26.36	1.02	23.05	0.995

BioC2.7/BSGenome	Raw DNA sequence from the Bioconductor package BSGenome, in compressed and uncompressed forms	rda	513	8.45	1.00	117.17	0.981
		
BioC2.7/BSGenome/u		un-packed	513	32.41	1.62	447.40	0.000

YaleTFBS/bedGraph4	Raw ChIP-seq data from the YaleTFBS dataset of the ENCODE project. Four different file types, both in compressed and uncompressed forms.	bed-Graph4	171	139.91	216.73	2447.62	0.924
		
YaleTFBS/bedGraph4.gz		bed-Graph4.gz	171	31.45	52.89	551.80	0.996
		
YaleTFBS/fastq		fastq	388	541.99	199.25	4469.89	0.919
		
YaleTFBS/fastq.gz		fastq.gz	388	160.75	49.55	1564.84	0.996
		
YaleTFBS/tagAlign		tagAlign	520	279.45	79.95	2357.32	0.544
		
YaleTFBS/tagAlign.gz		tag-Align.gz	520	96.70	27.86	815.63	0.994
		
YaleTFBS/wig		wig	33	10.66	188.92	693.66	0.912
		
YaleTFBS/wig.gz		wig.gz	33	3.27	59.76	207.93	0.996

Measurements of *δ*-variability in several biological datasets. Exact description of the experiment is available in the Supplementary material online [14].

For each dataset we found the byte-wise most similar pair of files, ignoring misnamings and differences in file sizes. The reported *δ *is the variability metric for this best pair (i.e. the proportion of pairwise different bytes). Exact details of this experiment as well as the data files are provided in the Supplementary Text online [14].

From the results we see that for most datasets, *δ *is at least 0.2, and for the majority of compressed datasets *δ *is at least 0.9. There seem to be some notable exceptions, however: the E61/fa, GPL570/cel, GPL570/cel.gz, BioC2.7/BSGenome/u datasets have at least a single pair of highly similar files. To better understand the implications of this result we examined the corresponding pairs manually (Table 2). Manual examination showed that for the non-DNA data used in our experiments, all cases of similar file pairs are instances of misformattings of the same raw data, which are irrelevant from the point of view of data analysis. In the case of DNA data, there were examples of a few pairs of DNA sequences which, when unpacked, are highly similar, as they correspond to different haplotypes or genome assemblies of the same organism.

**Table 2:** Detailed inspection of similar file pairs

Dataset	File pair and remarks	File sizes (in MB)	δ
E61/fa	Homo_sapiens.GRCh37.61.dna_rm.chromosome.HSCHR6_MHC_SSTO.fa	166.04	0.015
	Homo_sapiens.GRCh37.61.dna_rm.chromosome.HSCHR6_MHC_MANN.fa	166.06	
	These are two alternative haplotype "patch" files for the same chromosome locus. The dataset contains 11 other examples of similar file pairs with *δ *< 0.06 (when unpacked). All are related to the alternative haplotypes for the MHC locus. The next most similar pair of files has *δ *> 0.8.		

GPL570/cel	GSM405175.CEL	12.93	8e-6
	GSM341406.CEL	12.93	
	The second file differs from the first by a single Affymetrix probe measurement. According to GEO metadata the two files are simply different packagings of the same experimental data by two researchers. The GEO570 dataset contains 9 other examples of similar file pairs with *δ *< 0.002. The next most similar pair of files has *δ *> 0.3.		

GPL570/cel.gz	GSM405175.CEL.gz	4.31	6e-4
	GSM341406.CEL.gz	4.31	
	A gzip-compressed version of the pair above. Same remarks apply. The most similar pair of actually different datafiles has *δ *> 0.9.		

BioC2.7/B SGenome/u	BSgenome.Athaliana.TAIR.01222004/extdata/chr1.rda	29.04	2e-4
	BSgenome.Athaliana.TAIR.04232008/extdata/chr1.rda	29.04	
	Consequtive versions of A.thaliana reference genome. The next most similar file pair in this dataset has *δ *> 0.5. Note that the compressed versions of the same files have *δ *> 0.9.		

The table lists the suspiciously similar pairs of files from the studied datasets.

Those observations largely confirm the assumption of the wide applicability of PFFF hashing in the context of biological data. Care should be taken in the case of datasets containing *uncompressed genome sequences *with minor variations, when those variations *are key to the analysis*. In such situations it makes more sense to rely on consistent file naming, conventional hashing, or, best of all, compressed representation of the variations. As we can see in Table 1 even plain gzip compression is sufficient to ensure high *δ*-variability. Based on our measurements, we suggest *δ *= 0.9 as a reasonable choice for datasets consisting of compressed files and *δ *= 0.2 as a safe choice for general-purpose PFFF-hashing. Substituting *ε*_fail _= 2^-64 ^and *n *= 10^6 ^into Equation (1) we obtain that for *δ *= 0.9, a sample size of just ℓ = 32 blocks guarantees negligible probability of collisions. For *δ *= 0.2 the guarantees are satisfied by ℓ = 325.

### Algorithm performance

To compare PFFF to conventional hashing we compared the runtime of our algorithm to the (still) popular MD5 hashing algorithm in a variety of settings: hashing over HTTP and NFS network protocols, hashing on a local SSD (Flash) drive and on a portable USB hard disk. We should note that the choice of MD5 as the baseline is fairly arbitrary, as the hashing time is heavily dominated by I/O rather than actual hash value calculations.

**Figure 1:**
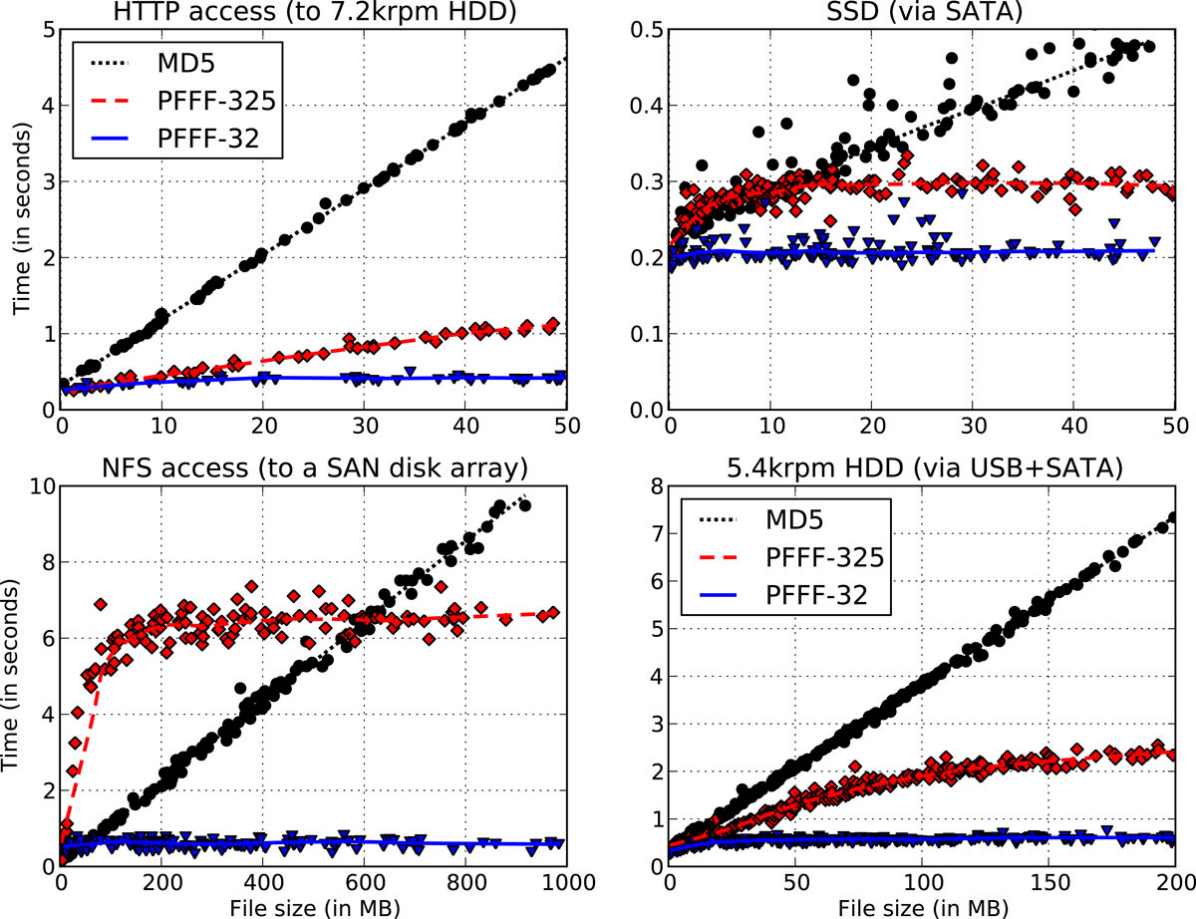
**Comparison of PFFF to conventional hashing**. The plots demonstrate time for hashing a single file of a given size by MD5 and by PFFF. Note that axis scales on the four plots are different.

In our experiments we considered the values of ℓ = 32 and ℓ = 325, corresponding to the two common situations highlighted in the previous section. Results are presented in Figure 1 and in the Supplementary Text [14]. Several interesting remarks are in order.

#### Performance of hashing via network

As one might expect, use of PFFF over HTTP outperforms conventional MD5 hashing for any file size (although for smaller file sizes the benefits are minor). The reason lies in the fact that HTTP protocol supports *range queries *[15]. This allows the client to make a single request demanding specifically those bytes which are necessary to compute the PFFF hash. This is much more efficient than a complete file download performed in the case of conventional hashing.

Another network protocol, NFS, however, demonstrates a different result. Contrary to HTTP, in case of NFS each of the samples has to be queried using a separate request. As a result, network latency starts dominating the processing time and in the case of ℓ = 325, PFFF hashing is actually *slower *than straightforward file scan for file sizes up to about 500MB (50MB for ℓ = 32). Of course, as file size grows larger, the timing of MD5 hashing continues to increase linearly while PFFF time stays constant, but for practical purposes of contemporary data analysis we must conclude that PFFF is not useful over NFS, not at least with the "safe" ℓ = 325 setting.

#### Performance on rotational and solid-state media

Random access to data on hard disk (HDD) and solid-state (SSD) drives does not come for free, as we can observe in the two rightmost panels of Figure 1. For file sizes up to about 10MB, timings of 325-sample PFFF are no better than that of a complete file scan. For larger files, PFFF becomes beneficial, but the situations in the case of SSD and HDD storage differ slightly. For SSD storage, the timing characteristic of PFFF flattens out fairly quickly (e.g. after reaching 0.3 seconds at 10MB for ℓ = 325). For a rotational hard drive, however, timing continues to grow slowly with file size until for ℓ = 325 it plateaus somewhere around 2.5 seconds for file sizes of 200MB or larger.

The main reason for this effect lies in the operation of a rotational drive. On such drives, data is accessed via continuously rotating magnetic heads reading data off *tracks*. For smaller files, multiple PFFF samples can often happen to be located on the same track and thus read within a single revolution of the disk. As file size becomes large enough, however, each byte request will typically require a track switch followed by a seek operation. Such an operation requires, on average, half a revolution. As the time per revolution for a 5400rpm drive is approximately 11ms, randomly accessing 325 samples requires about 1.8 seconds. Our actual measured time is just slightly larger as it also includes file system access and USB communication overhead.

In general, assuming that a typical rotational hard drive can read as much as 0.5M of data in a single revolution, we can estimate that for sufficiently large files, an ℓ-sample PFFF hash computation requires approximately as much time as a full scan of a 0.25ℓ-megabyte file.

Note that we observe the same effect in our HTTP experiment for ℓ = 325, due to the fact that the HTTP server was also using a HDD backend.

This observation limits the usefulness of PFFF for rotational media to some extent. The situation may be alleviated by using a small number of large blocks instead of a large number of single bytes in PFFF hashing. In fact, our measurements confirm that among all the files in our datasets except for uncompressed haplotype variations, no two distinct files (misformattings not taken into account) share even a single common block of size 0.5M, which means that *δ *for such a large block size is close to 1.

### Application in duplicate detection

An interesting alternative application for PFFF is fast detection of unwanted duplicates or format errors in large scientific data warehouses. Intuitively, if two distinct files yield the same PFFF hash value then they are either identical or highly similar and thus deserve further investigation.

More formally, we can regard duplicate detection as a classical statistical hypothesis testing problem, where the null hypothesis states that all the *n *files in a data warehouse satisfy the *δ*-variability condition. We can now fix *ε*_fail _= 0.05 (a significance level threshold commonly used in hypothesis testing), compute the corresponding ℓ value using Equation (1) and apply PFFF with this value of ℓ to all files in the warehouse. Any hash collisions can now be regarded as evidence, rejecting the null hypothesis.

To provide a more specific example, consider the GPL570/cel.gz dataset from the GEO warehouse, listed in Table 1. Considering that it consists of gzip-compressed files we can postulate a true *δ*-variability between meaningfully distinct files of at least 0.9. Substituting *δ *= 0.9, *n *= 59892, *ε*_fail _= 0.05 into Equation (1) we obtain ℓ ≈ 11. Having applied PFFF with ℓ = 11 on the files of the dataset we discovered 8165 groups of equal or equivalent files, the largest of them comprising 10 files. The total volume of redundant files was more than 54 gigabytes, unnecessarily hogging more than 16% of the dataset space!

### Implementation details

The implementation of our algorithm is available as an open-source tool **pfff **available for download at http://biit.cs.ut.ee/pfff. Besides the basic probabilistic hash function, the tool provides several additional convenience features, such as the ability to include file headers and file size in the hash. The whole package is available under the open source BSD License, both as as stand alone tool and a C library.

## Discussion

The main requisite for PFFF is that the data to be analysed must satisfy the variability assumption. Biological data sets are likely to satisfy this requirement for two reasons. First, there is inherent variability in the biological systems - no two cells are identical. Secondly, even if the measurements are taken from the same cell at the same time point, the complicated measurement procedures result in sufficient amount of measurement errors. It is highly unlikely to obtain *exactly *the same results in two different experiments. Nonetheless, situations are possible, where the level of variability is rather low. For instance, raw sequencing data of individuals has low variance, as only 2-5% of base pairs differ from the reference sequence. In such cases, it is normally more advantageous to store and transfer differences (SNP's, insertions and deletions) to save the storage space and network load. Such compression increases variability and enables the application of PFFF. Other file compression techniques (e.g. gzip) have roughly the same effect, as they remove repeating patterns that are shared over files.

In addition, PFFF's reliability may be boosted to near certainty for all practical purposes by including into the fingerprint the size of the file and the first megabyte or so of data. Indeed, in most file formats the *header *usually contains most of the important identifying information.

In our experiments we discovered that despite the theoretical guarantees of constant runtime for PFFF, several practical aspects, such as network latency, rotational operation of hard disks and sequential access optimizations implemented on the operating system level (e.g. data prefetching), may limit the advantages of the approach over conventional hashing. We observed that the benefits of PFFF hashing are strongest when data must be accessed over HTTP or when SSD storage is involved. In other contexts, application of PFFF requires further assumptions in order to provide significant advantages.

In particular, for very large files (with sizes approaching and exceeding a gigabyte) PFFF is nearly always meaningful. Alternatively, higher *δ*-variability in the data allows to reduce the ℓ parameter, drastically reducing the number of requests. As we noted, *δ *= 0.9 and the corresponding ℓ = 32 are meaningful settings for collections of compressed files.

Higher *δ*-variability can also often be assumed by increasing block size. Moreover, for rotational storage, meaningful block sizes are anyway on the order 0.5 megabytes, as this is the the chunk of data read by the disk during a single revolution.

Finally, note that the need for our method would be greatly diminished if the prominent warehouses agreed on publishing deterministic hashes together with the data files. Unfortunately, it may still take a few years before such agreement emerges. After that, the PFFF hash could still provide a useful fallback mechanism.

## Conclusion

We have proposed a specially tailored method for fingerprinting biological data files, which can significantly reduce consumption of critical resources in several common usage patterns of big data analysis workflows. Our PFFF algorithm allows for rapid checking of equivalence of large files acquired from biological samples with little computational overhead, thus possibly reducing the number of files read and transferred between and within data centers.

The application of PFFF is not limited to biological data. Indeed, probabilistic fingerprinting applies to any data with high variability. This includes, in particular, data that contains noisy measurements, such as scientific data in astronomy or particle physics, as well as sound and video files.

## Competing interests

The authors declare that they have no competing interests.

## Authors' contributions

PP conceived the idea of the approach and drafted the manuscript. SL developed the idea theoretically and helped draft the manuscript. KT developed the idea theoretically, implemented the algorithm, performed experiments, prepared figures and supplementaries, and wrote the final manuscript. GS and JV participated in the coordination of the study and helped draft the manuscript. All authors read and approved the final manuscript.

## Declarations

The publication costs for this article were funded by the European Regional Development Fund through the Estonian Center of Excellence in Computer Science, EXCS.

This article has been published as part of *BMC Genomics *Volume 14 Supplement 2, 2013: Selected articles from ISCB-Asia 2012. The full contents of the supplement are available online at http://www.biomedcentral.com/bmcgenomics/supplements/14/S2.

## References

[B1] Doctorow C Big data: Welcome to the petacentre Nature 2008 455 7209 16 21 http://dx.doi.org/10.1038/455016a 10.1038/455016a 18769411

[B2] Richter BG Sexton DP Managing and analyzing next-generation sequence data PLoS Comput Biol 2009 5 6 e1000369 10.1371/journal.pcbi.1000369 19557125 PMC2667638

[B3] Tridgell A Efficient algorithms for sorting and synchronization PhD thesis 1999 The Australian National University http://www.samba.org/~tridge/phd_thesis.pdf

[B4] Carter LJ Wegman MN Universal classes of hash functions Proceedings of the ninth annual ACM symposium on Theory of computing 1977 New York, NY, USA: ACM 106 112 http://dx.doi.org/10.1145/800105.803400

[B5] Trachtenberg A Starobinski D Agarwal S Fast PDA synchronization using characteristic polynomial interpolation INFOCOM 2002 3 1510 1519 http://people.bu.edu/staro/infocom02pda.pdf

[B6] Minsky Y Trachtenberg A Zippel R Set reconciliation with nearly optimal communication complexity IEEE Transactions on Information Theory 2003 49 9 2213 2218 10.1109/TIT.2003.815784

[B7] Parkinson H Kapushesky M Shojatalab M Abeygunawardena N Coulson R Farne A Holloway E Kolesnykov N Lilja P Lukk M Mani R Rayner T Sharma A William E Sarkans U Brazma A ArrayExpress-a public database of microarray experiments and gene expression profiles Nucleic Acids Res 2007 35 Database D747 D750 10.1093/nar/gkl995 17132828 PMC1716725

[B8] Barrett T Troup DB Wilhite SE Ledoux P Evangelista C Kim IF Tomashevsky M Marshall KA Phillippy KH Sherman PM Muertter RN Holko M Ayanbule O Yefanov A Soboleva A NCBI GEO: archive for functional genomics data sets-10 years on Nucleic Acids Res 2011 39 Database D1005 D1010 10.1093/nar/gkq1184 21097893 PMC3013736

[B9] Rivest R The MD5 Message-Digest Algorithm 1992 US

[B10] National Institute of Standards and Technology FIPS 180-3, Secure Hash Standard, Federal Information Processing Standard (FIPS), Publication 180-3 2008 Tech. rep., Department of Commerce

[B11] Menezes AJ van Oorschot PC Vanstone SA Handbook of Applied Cryptography 2001 CRC Press http://www.cacr.math.uwaterloo.ca/hac/

[B12] Carter JL Wegman MN Universal classes of hash functions (Extended Abstract) STOC '77: Proceedings of the ninth annual ACM symposium on Theory of computing 1977 New York, NY, USA: ACM 106 112

[B13] Matsumoto M Nishimura T Mersenne twister: a 623-dimensionally equidistributed uniform pseudo-random number generator ACM Trans Model Comput Simul 1998 8 3 30 10.1145/272991.272995

[B14] PFFF: Supplementary text, materials, software and code (online) http://biit.cs.ut.ee/pfff/

[B15] Fielding R Gettys J Mogul J Frystyk H Masinter L Leach P Berners-Lee T Hypertext Transfer Protocol-HTTP/1.1 RFC 2616 (Draft Standard) 1999 Internet Engineering Task Force http://www.ietf.org/rfc/rfc2616.txt Updated by RFCs 2817, 5785, 6266, 6585

